# Antineoplastic Effects of α-Santalol on Estrogen Receptor-Positive and Estrogen Receptor-Negative Breast Cancer Cells through Cell Cycle Arrest at G2/M Phase and Induction of Apoptosis

**DOI:** 10.1371/journal.pone.0056982

**Published:** 2013-02-22

**Authors:** Sreevidya Santha, Ajay Bommareddy, Brittny Rule, Ruth Guillermo, Radhey S. Kaushik, Alan Young, Chandradhar Dwivedi

**Affiliations:** 1 Department of Pharmaceutical Sciences, South Dakota State University, Brookings, South Dakota, United States of America; 2 Department of Pharmaceutical Sciences, Wilkes University, Wilkes-Barre, Pennsylvania, United States of America; 3 Department of Biology and Microbiology, South Dakota State University, Brookings, South Dakota, United States of America; 4 Department of Veterinary and Biomedical Sciences, South Dakota State University, Brookings, South Dakota, United States of America; II Università di Napoli, Italy

## Abstract

Anticancer efficacy and the mechanism of action of α-santalol, a terpenoid isolated from sandalwood oil, were investigated in human breast cancer cells by using p53 wild-type MCF-7 cells as a model for estrogen receptor(ER)-positive and p53 mutated MDA-MB-231 cells as a model for ER-negative breast cancer. α-Santalol inhibited cell viability and proliferation in a concentration and time-dependent manner in both cells regardless of their ER and/or p53 status. However, α-santalol produced relatively less toxic effect on normal breast epithelial cell line, MCF-10A. It induced G2/M cell cycle arrest and apoptosis in both MCF-7 and MDA-MB-231 cells. Cell cycle arrest induced by α-santalol was associated with changes in the protein levels of BRCA1, Chk1, G2/M regulatory cyclins, Cyclin dependent kinases (CDKs), Cell division cycle 25B (Cdc25B), Cdc25C and Ser-216 phosphorylation of Cdc25C. An up-regulated expression of CDK inhibitor p21 along with suppressed expression of mutated p53 was observed in MDA-MB-231 cells treated with α-santalol. On the contrary, α-santalol did not increase the expression of wild-type p53 and p21 in MCF-7 cells. In addition, α-santalol induced extrinsic and intrinsic pathways of apoptosis in both cells with activation of caspase-8 and caspase-9. It led to the activation of the executioner caspase-6 and caspase-7 in α-santalol-treated MCF-7 cells and caspase-3 and caspase-6 in MDA-MB-231 cells along with strong cleavage of poly(ADP-ribose) polymerase (PARP) in both cells. Taken together, this study for the first time identified strong anti-neoplastic effects of α-santalol against both ER-positive and ER-negative breast cancer cells.

## Introduction

α-Santalol is a naturally occurring terpenoid isolated from sandalwood tree (*Santalum album* Linn) [1]. Both the wood and oil produce a distinctive fragrance which has been highly valued for centuries. The essential oil, emulsion and paste of sandalwood have been traditionally used in the treatment of various diseases in some parts of the world, also used in food industry as a flavor ingredient and topically in cosmetics and perfumes [1], [2]. The efficacy of α-santalol as a chemopreventive agent appears to be very promising in skin cancer control [3]–[5]. Previous studies from our laboratory have shown excellent chemopreventive effects of α-santalol against 7,12-dimethylbenzanthracene (DMBA) initiated and 12-O-tetradecanoylphorbol-13-acetate (TPA) induced skin tumorigenesis in CD-1 and SENCAR mice [3] and ultraviolet-B induced skin tumorigenesis in SKH-1 hairless mice [4]. Treatment with α-santalol appears to be non-toxic to normal tissues over a wide range of concentrations. We recently reported the antineoplastic effects of α-santalol on human prostate cancer cell lines which are either androgen independent (PC-3) or androgen dependent (LNCaP) [6]. Despite these studies on skin cancer and prostate cancer models, the efficacy of α-santalol on other types of cancer has not been explored. In this study we have investigated the anticancer effects and mechanisms of action of α-santalol on human breast cancer cells by using MCF-7 cells (p53 wild type) as a model for estrogen receptor (ER)-positive and MDA-MB-231 cells (p53 mutant) as a model for ER-negative breast cancer.

Despite significant advances in therapeutic, early detection and diagnostic strategies, the incidence and mortality rates of breast cancer are still increasing. Patients with ER-positive breast cancer generally have a better prognosis and are more likely to respond to hormonal therapy; but ER-negative breast cancer is more aggressive and unresponsive to anti-estrogens [7]. Treatment options for ER-negative breast cancer patients are limited to conventional cytotoxic chemotherapy, which is not effective in the advanced stages. [8]–[10]. Moreover, hormone therapy and chemotherapy are not completely effective due to its non-specific mechanisms of action, and the presence of resistant cancer cells [11], [12]. Also, long-term treatment with tamoxifen leads to a higher risk for the development of endometrial cancer [13]. Hence, it is important to develop more effective and safer chemopreventive agents to control both ER-positive and ER-negative breast cancers.

This study for the first time identified strong anti-neoplastic effects of α-santalol against both ER-positive and ER-negative breast cancer cells. α-Santalol inhibited cell viability and proliferation, caused G2/M cell cycle arrest and induced apoptotic cell death through extrinsic and intrinsic pathways in both cell lines. However, α-Santalol produced relatively less toxic effect on normal breast epithelial cell line MCF-10A. Further mechanistic studies have identified alterations of various proteins that are involved in α-santalol mediated apoptotic cell death and G2/M cell cycle arrest which further elucidates the mechanisms of anti-neoplastic effects of α-santalol on breast cancer.

## Materials and Methods

### Reagents

Cleaved caspase-3, -6, -8, Cleaved poly(ADP-ribose) polymerase (PARP), BRCA1 and Chk1 antibodies were obtained from Cell Signaling Technology (Beverly, MA). Cyclin-B1 antibody was from Millipore (Billerica, MA). Caspase-7 p20 antibody, Caspase-9, Cyclin-A, CDK2, Cdc2, Cdc25B, Cdc25C, Pcdc25C (Ser216), p53, p21, β-actin and secondary antibodies were from Santa Cruz Biotechnology (Santa Cruz, CA). Dulbecco's modified eagle's medium (DMEM), Fetal bovine serum (FBS), Penicillin-streptomycin solution, trypsin EDTA and phosphate buffered saline (PBS) were from Mediatech, Inc. (Herndon, VA). MEGM™ Mammary Epithelial Cell Growth Medium Bullet Kit was from Lonza/Clonetics (Walkersville, MD.) Cholera toxin was from Sigma (St. Louis, MO). Other reagents were obtained in their highest purity grade available commercially.

### Cell Culture

Human breast cancer cell lines MCF- 7 and MDA-MB-231 and nonmalignant human mammary epithelial cell line MCF-10A (ATCC, Manassas, VA) were grown under standard culture conditions at 37°C in a humidified atmosphere containing 5% CO2. MCF-7 and MDA-MB-231 cells were maintained in DMEM supplemented with 10% FBS, 100 unit/ml of penicillin and 100 µg/ml of streptomycin. Nonmalignant human mammary epithelial cell line, MCF-10A, was maintained in MEGM (Lonza) that contained all the growth supplements that were provided with the kit, 5% horse serum and 100 ng/ml cholera toxin. For each of the experiments, the cells were treated with different concentrations of α-santalol or with solvent (DMSO) in an equivalent amount that is the highest used for the treatment.

### Isolation of α-Santalol

α-Santalol was isolated from sandalwood oil (Organic Infusions, Inc) by column chromatography with n-Hexane: Ethyl acetate 3∶1 as a solvent system and the purity was assessed by gas chromatography [14].

### Cell Viability Assay

Cells were plated at a density of 5000 cells/well in 96-well plate and allowed to attach overnight. Next day, cells were treated with media containing DMSO alone as control or with 10–100 µM α-santalol in DMSO for 12, 24 and 48 h. Cell viability was determined at the end of each treatment by 3-(4,5-dimethylthiazol-2-yl)-2,5-diphenyltetrazolium bromide (MTT) assay as previously described [15]. A blank reading was subtracted from experimental readings for final calculations.

### BrdU Incorporation Assay for Cell Proliferation

BrdU cell proliferation ELISA was used to quantitate cell proliferation based on the measurement of BrdU incorporation during DNA synthesis in proliferating cells by using a colorimetric BrdU cell proliferation ELISA kit (Roche Applied Science, IN, USA). Cells were seeded and treated as for MTT assay and proliferation rate was quantified according to manufacturer’s instruction as described earlier [5]. A blank reading was subtracted from experimental readings before final calculations.

### Quantitation of DNA Fragmentation by TUNEL Assay and Flow Cytometry

Apo-BrdU TUNEL assay kit (Invitrogen) was used to quantify the amount of DNA fragmentation in α-santalol treated cells as per manufacturer's protocol. Briefly, cells were treated with either DMSO as control or with 50–100 µM α-santalol for 48 h. Cells were collected by trypsinization and fixed with 1% paraformaldehyde followed by 70% ethanol. The fragmented DNA in apoptotic cells were labeled with BrdU followed by incubation with Alexa Fluor 488 dye-labeled anti-BrdU antibody. The stained cells were analyzed by flow cytometry and the extent of DNA fragmentation was quantified by a computational analysis of cells staining positive for BrdU, using Cellquest software.

### Cell Cycle Analysis

MCF-7 and MDA-MB-231 cells were treated with 25–75 µM α-santalol or media with DMSO as control for 12 and 24 h. Treated cells were collected by trypsinization and fixed with 70% ethanol in PBS. Cells were washed with PBS and suspended in 250 µl of PBS and treated with 100 µl of RNase A (1 mg/ml) for 30 min at 37°C followed by staining with 40 µg/ml propidium iodide (PI) in the dark at room temperature for 30 min. The samples were analyzed with BD FACScan™ flow cytometry (BD Biosciences, San Jose, CA) using CellQuest software to determine cell cycle distribution.

### Immunoblotting

To determine the effect of α-santalol on proteins of interest, whole cell lysates were prepared and supernatants were collected with micro-centrifugation. Protein concentration in each sample was determined by BCA™ protein assay kit (Pierce, Rockford, IL) with albumin as a standard. Equal amounts of proteins were denatured and separated on polyacrylamide gels. The proteins in the gels were transferred on to nitrocellulose membranes and probed with appropriate primary antibodies followed by appropriate horseradish peroxidase (HRP) conjugated secondary antibody. Protein expression was detected by enhanced chemiluminescence (ECL Plus detection kit, Amersham Biosciences, NJ) by using a UVP Biochem Gel Documentation system (UVP Inc, Upland, CA). The expression of β -actin was used as a loading control.

### Statistical Analysis

ANOVA followed by Tukey's post test was applied to compare the statistical significance of the difference between different treatment groups and control. Significance was considered at p<0.05.

## Results

### α-Santalol Inhibits Cell Viability in ER-positive and ER-negative Breast Cancer Cells

**Figure 1:**
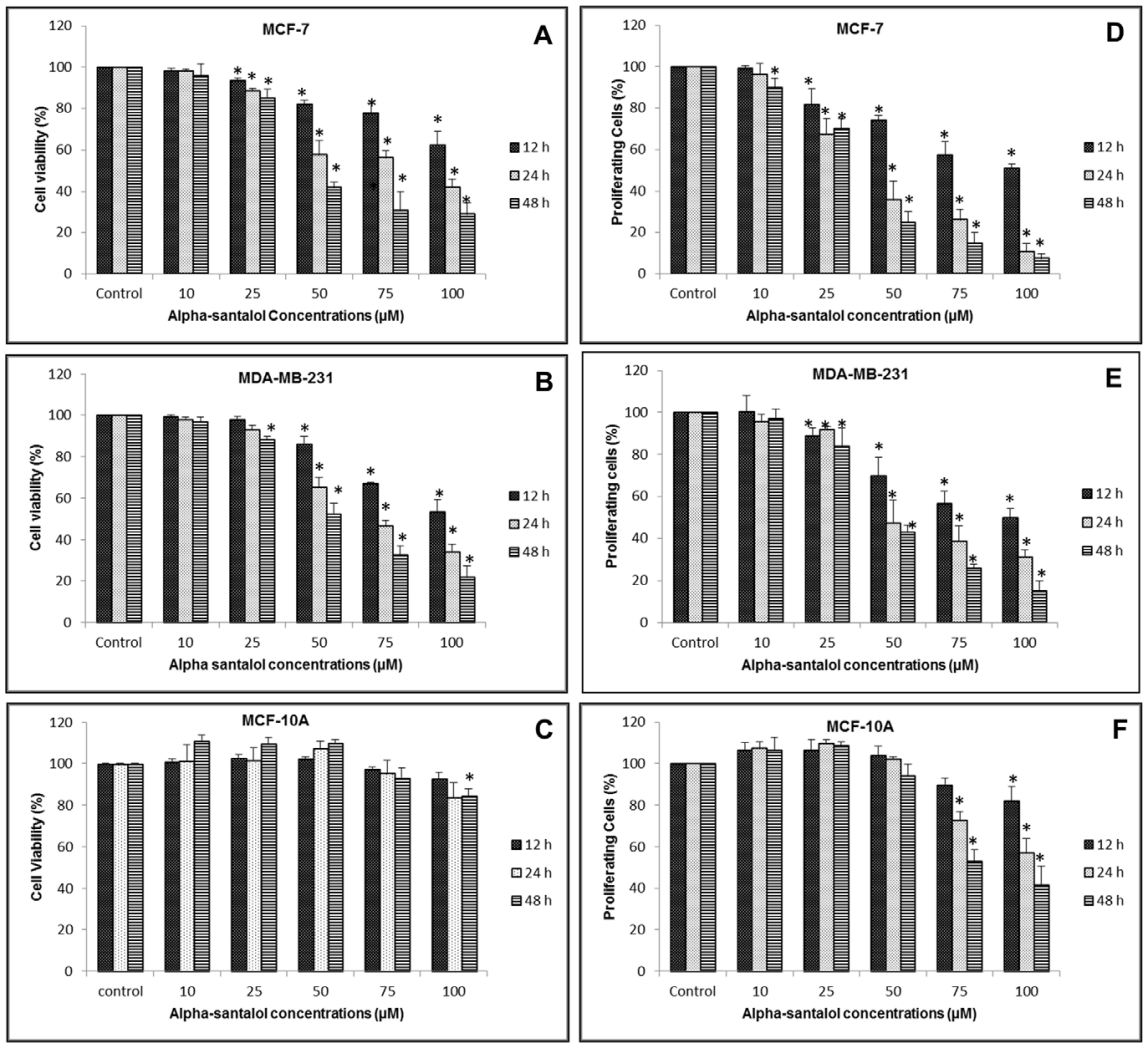
Effect of α-santalol on cell viability and proliferation. Human breast cancer cells MCF-7 and MDA-MB-231 and normal human breast epithelial cells MCF-10A were treated with either DMSO (control) or 10–100 µM α-santalol for 12, 24 and 48 h. At the end of respective treatments, MTT and BrdU incorporation assays were performed on each cell line. Data in left panels (A–C) were obtained from MTT assays and data in right panels (D–F) were from BrdU cell proliferation ELISA of MCF-7, MDA-MB-231 and MCF-10A cells respectively. Values were shown as mean ± SD of at least three experiments. *, P<0.05 indicates statistically significant decrease in α-santalol treated groups as compared with the control.

To explore the anticancer effect of α-santalol against human breast cancer, we initially conducted MTT cell viability assays in breast cancer cells which are ER-positive (MCF-7) as well as ER-negative (MDA-MB-231) and in a normal breast epithelial cell line (MCF-10A). As shown in Figure 1A and 1B, α-santalol treatment resulted in a reduction in cell viability in a time and concentration-dependent manner in MCF-7 and MDA-MB-231 cells. At 10–100 µM concentrations, α-santalol resulted in 2–38%, 2–58% and 4–71% reduction in cell viability in MCF-7 cells and 1–47%, 2–66% and 4–79% in MDA-MB-231 cells after 12, 24 and 48 h of treatments respectively. On the other hand, the normal breast epithelial cell line MCF-10A was found more resistant, and reduction in cell viability was observed only with higher concentrations of α-santalol in it (Figure 1C).

### α-Santalol Inhibits Cell Proliferation in ER-positive and ER-negative Breast Cancer Cells

For further confirmation of the antineoplastic activity of α-santalol against human breast cancer, we performed BrdU cell proliferation ELISA. Like MTT assay, α-santalol treatment resulted in a concentration and time-dependent reduction in cell proliferation rate in MCF-7 and MDA-MB-231 cells (Figures 1D and 1E). The percentage decrease of cell proliferation was 1–49.5%, 4–89% and 10–93% at 10–100 µM concentrations of α-santalol in MCF-7 and 0–50%, 4–69% and 3–85% in MDA-MB-231 cells after 12, 24 and 48 h of treatments. In MCF-10A cells, α-santalol treatment resulted in a significant reduction (p<0.05) of cell proliferation only at higher concentrations (Figure 1F).

### α-Santalol Induced DNA Fragmentation in Breast Cancer Cells

**Figure 2:**
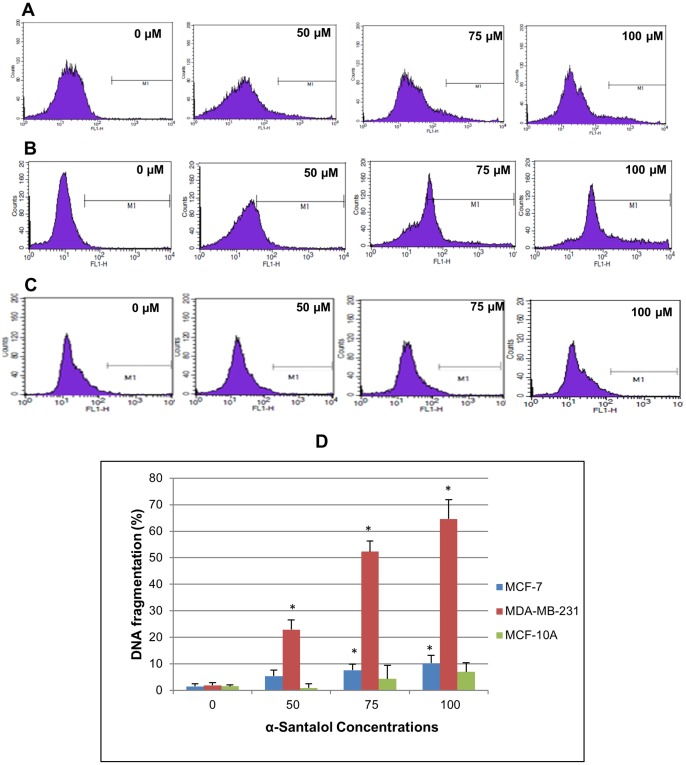
Effect of α-santalol on DNA fragmentation by TUNEL assay and flow cytometry. (A) MCF-7, (B) MDA-MB-231 and (C) MCF-10A cells were treated with α-santalol (0–100 µM) for 48 h and the extent of DNA fragmentation was determined by flow cytometric analysis. APO-BrdU TUNEL assay kit (Invitrogen) was used for the experiment and BrdU incorporation at DNA strand breaks of apoptotic cells were detected by conjugation to an Alexa Fluor 488 dye-labeled anti-BrdU antibody. The extent of DNA fragmentation was quantified by computational analysis of cells staining positive for BrdU using CellQuest software. (D) The bar graph indicates the percentages of apoptotic cells with fragmented DNA in MCF-7, MDA-MB-231 and MCF-10A cells. In each case data represents mean ± SD of three observations. *, P<0.05 indicates statistical significance in α-santalol treated groups compared with the control.

To assess whether α-santalol induce fragmentation of DNA that results from apoptotic signaling cascades, and which is a hallmark of late-stage apoptosis [16], we performed TUNEL assay after treating the cells with 50–100 µM of α-santalol for 48 h. Treatment with α-santalol induced strong DNA fragmentation in MDA-MB-231 cells when compared to MCF-7 and MCF-10A cells (Figures 2A–2C). The percentage DNA fragmentation induced by α-santalol in different treatment groups and control in MCF-7, MDA-MB-231 and MCF-10A cells are shown in Figure 2D. Our data indicate that when compared to inhibition of cell viability and proliferation as determined by MTT and BrdU ELISA, the percentage of DNA fragmentation resulted from apoptotic cell death is much lower in MCF-7 cells even after 48 h treatment of α-santalol. In MDA-MB-231 cells, the induction of DNA fragmentation was significant at all concentrations used and it resulted in about five times more DNA fragmentation when compared to MCF-7 cells. The percentage DNA fragmentation was lowest in normal breast epithelial cell line (MCF-10A) when compared to ER-positive (MCF-7) and ER-negative (MDA-MB-231) breast cancer cells. Since α-santalol produced a strong antineoplastic effect on both ER-positive and ER-negative breast cancer cells as compared to that on normal breast epithelial cells, further experiments were conducted on MCF-7 and MDA-MB-231 cells to explore the mechanisms of its action.

### α-Santalol Induced G2/M Phase Cell Cycle Arrest in MCF-7 and MDA-MB-231 Cells

**Figure 3:**
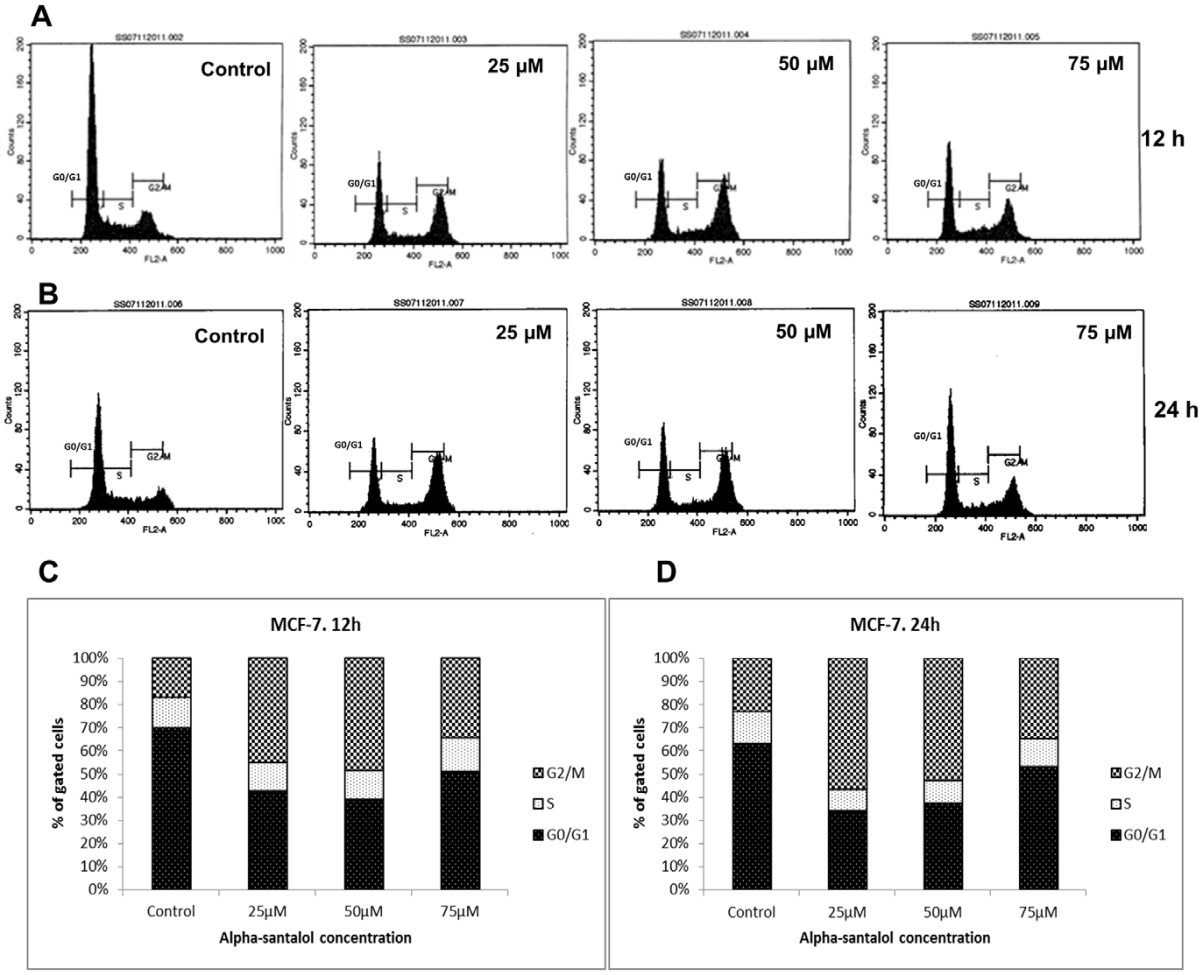
Effect of α-santalol on cell cycle progression in MCF-7 cells. Cells were treated with DMSO (control) or 25–75 µM of α-sanatlol for 12 and 24 h, stained with propidium iodide and distribution of cells in different phases of cell cycle were analyzed by flow cytometer. Histograms representing the fluorescence pattern for cell cycle distribution in different treatments are shown for 12 h (A) and 24 h (B). The percentage of cells in each cell cycle phases after 12 h and 24 h respectively are shown in (C) and (D). Data shown here are representative of those having similar results.

**Figure 4:**
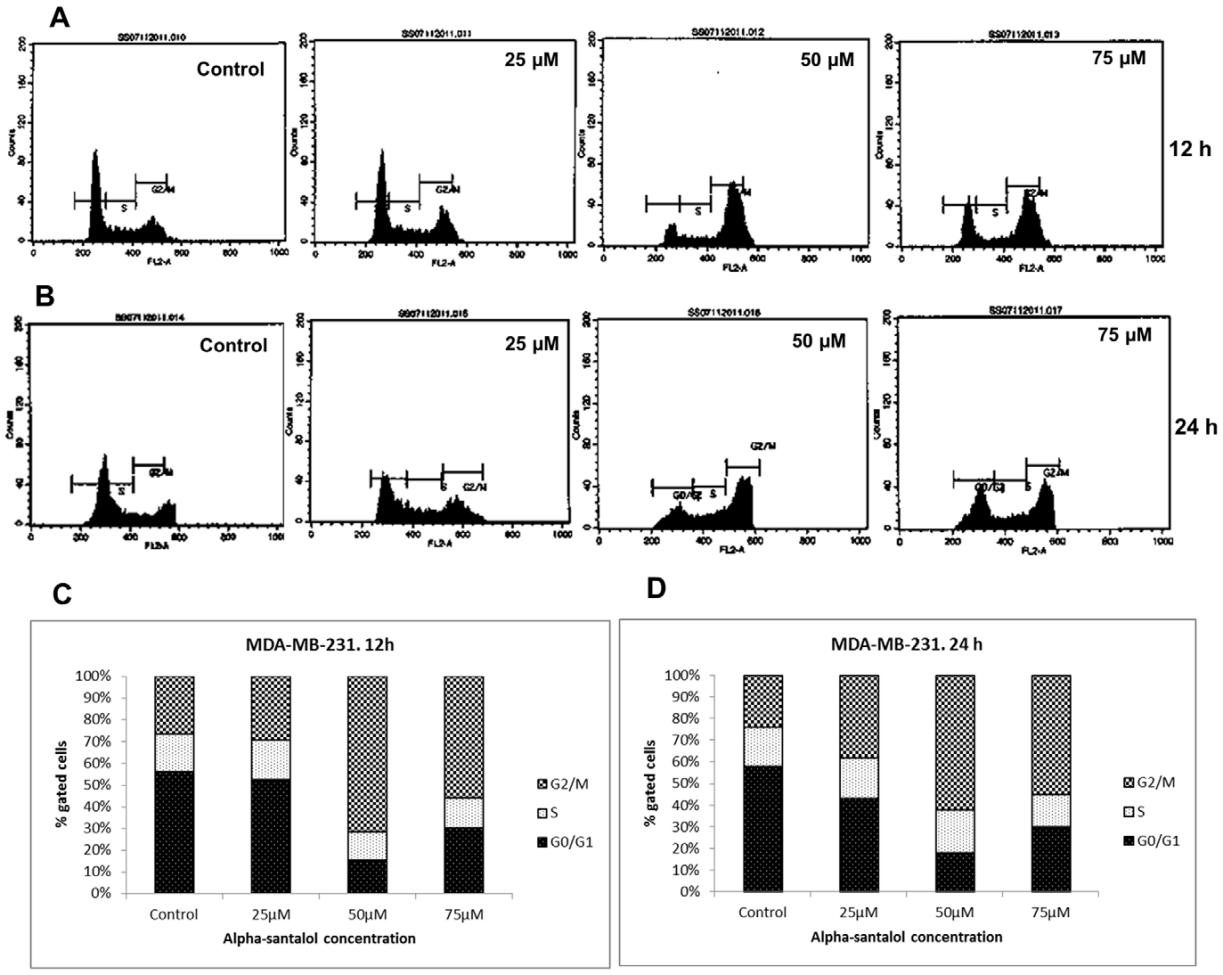
Effect of α-santalol on cell cycle progression in MDA-MB-231 cells. Data were obtained as explained in figure 3. Representative histograms of the fluorescence pattern for cell cycle distribution in different treatments after 12 h (A) and 24 h (B) are shown. (C) and (D**)** are the percentages of MDA-MB-231 cells in each cell cycle phases after 12 and 24 h α-santalol treatment.

In order to understand the underlying mechanisms of inhibition of cell viability and proliferation by α-santalol treatment, we analyzed its effects on cell cycle phase distribution in MCF-7 and MDA-MB-231 cells. As shown in Figures 3A and 3B, α-santalol induced strong accumulation of cells in the G2/M phase of the cell cycle in MCF-7 cells at all concentrations after 12 and 24 h treatments. This accumulation of cells in G2/M phase caused by α-santalol was associated with parallel depletion of cells in the G0/G1 phase. We observed almost similar findings in MDA-MB-231 cells (Figures 4A and 4B), except that the difference in G2/M population at 25 µM for 12 h treatment was not significantly different when compared to control. The percentage distributions of cells in each phases of cell cycle after 12 and 24 h α-santalol treatments are shown in Figures 3C & 3D (MCF-7) and Figures 4C & 4D (MDA-MB-231). Our findings suggest that α-santalol induced a strong G2/M phase arrest in breast cancer cells regardless of their ER or p53 status.

### α-Santalol Induces Activation of Multiple Caspases and PARP Cleavage in MCF-7 and MDA-MB-231 Cells

**Figure 5:**
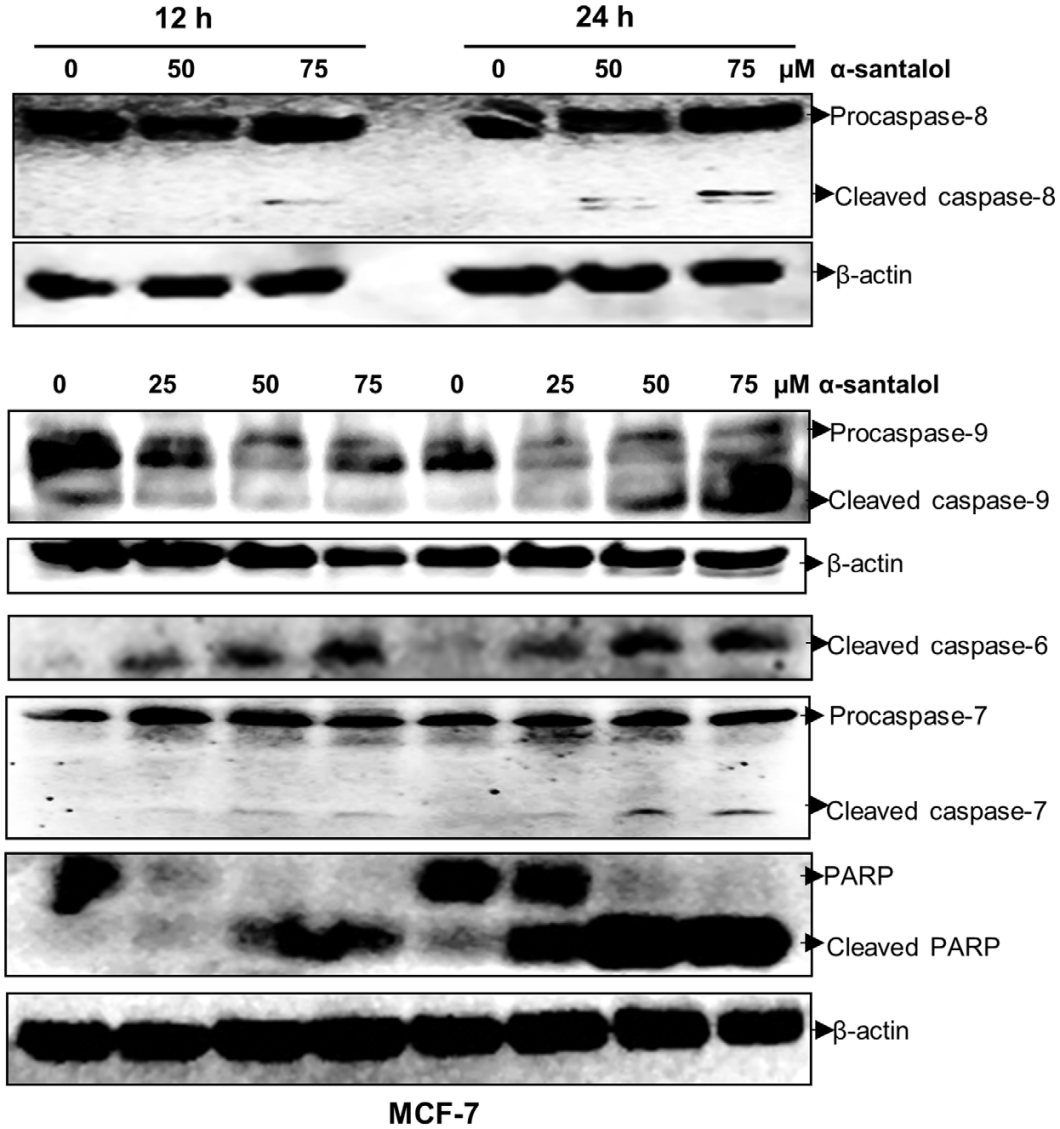
α-Santalol induces activation of different caspases and PARP cleavage in MCF-7 cells. Total cell lysates were prepared from the cells treated with the indicated concentration of α-sanatlol for 12 and 24 h. Equal amounts of proteins were separated by SDS-PAGE and subjected to western immunoblotting. Membranes were probed with respective primary antibodies followed by appropriate secondary antibody and protein expression was determined by ECL detection system. β-actin was used to verify equal loading of the samples.

**Figure 6:**
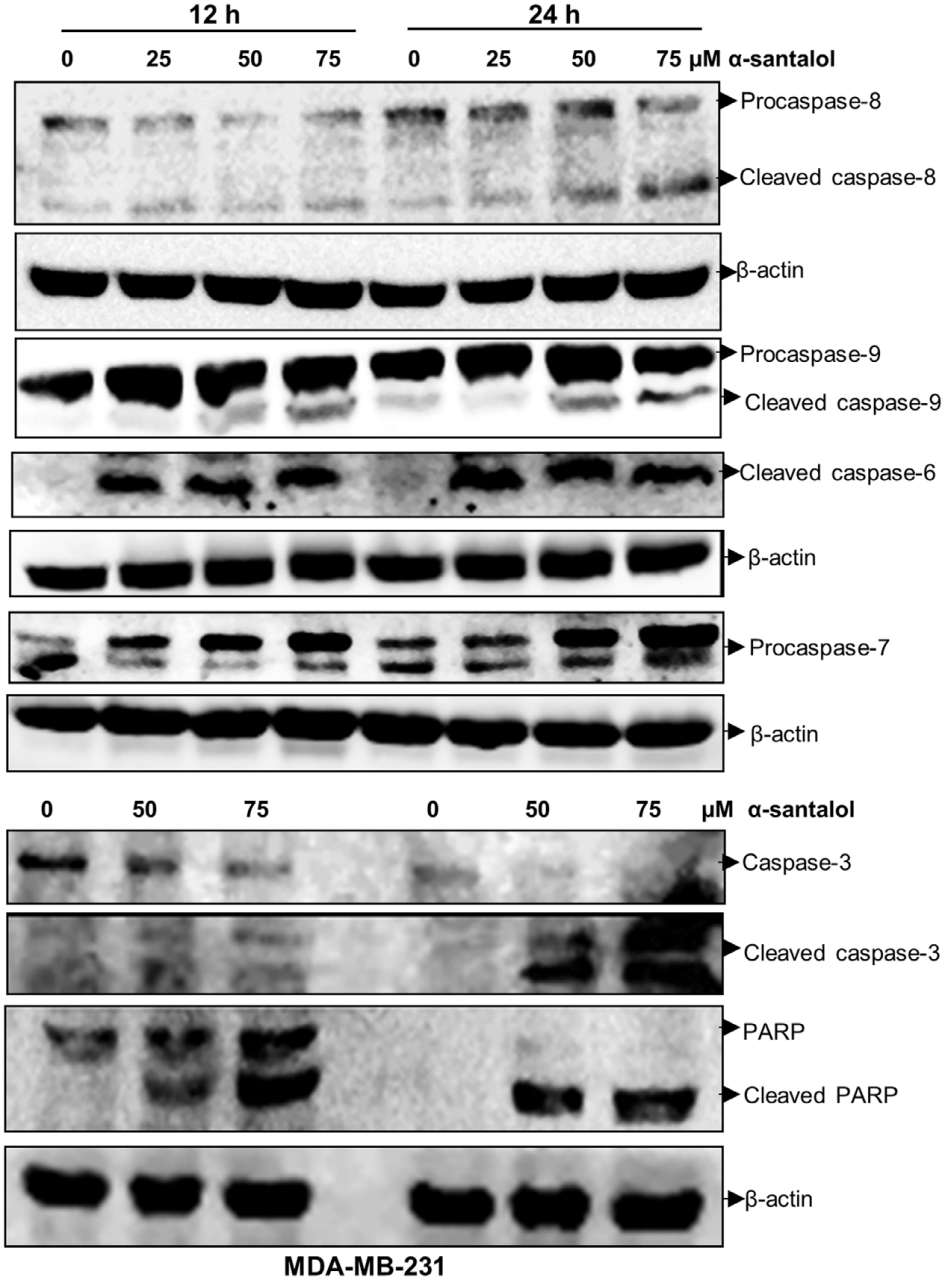
α-Santalol induces apoptosis in MDA-MB-231 cells by activating different caspases and PARP cleavage. Total cell lysates were made and the expressions of various apoptotic proteins were determined by western immunoblotting. β-actin was used as an internal control.

We examined the activation of different caspases and cleavage of PARP, which are hallmarks of apoptosis, and the results are shown in Figure 5 (MCF-7) and Figure 6 (MDA-MB-231). Our western blotting data suggests the involvement of both extrinsic and intrinsic pathways of apoptosis in MCF-7 and MDA-MB-231 cells as observed by the activation of both caspase-8 and caspase-9. In apoptotic pathways activation of these initiator caspases leads to the activation of executioner caspase such as caspase-3, -6, and -7, which then leads to the cleavage of proteins such as DNA repair enzyme, PARP. In MCF-7 cells treatment with α-santalol induces the activation of caspase-6 and caspase-7, and in MDA-MB-231 cells it induces the activation of caspase-3 and caspase-6. In MDA-MB-231 cells we found two adjacent bands at the procaspase-7 region (28–38 kDa), one of which showed an increase and the second one showed a decrease with α-santalol treatment. But the active p20 subunit was absent in MDA-MB-231 cells, which was observed in MCF-7 cells upon α-santalol treatment. In addition, we could not detect the expression of caspase-3 in control or α-santalol treated MCF-7 cells, whereas, time and concentration dependent activation of caspase-3 was observed in MDA-MB-231 cells. Western blot analysis of PARP showed the cleavage of PARP protein with concomitant reduction of 116 kDa full-length fragment, and accumulation of the 89 kDa cleaved fragment in both cells. All these data provide the evidence for the induction of apoptosis in breast cancer cells by α-santalol treatment.

### α-Santalol Changed Expression of Proteins Involved in the G2/M Phase Transition in MCF-7 and MDA-MB-231 Cells

**Figure 7:**
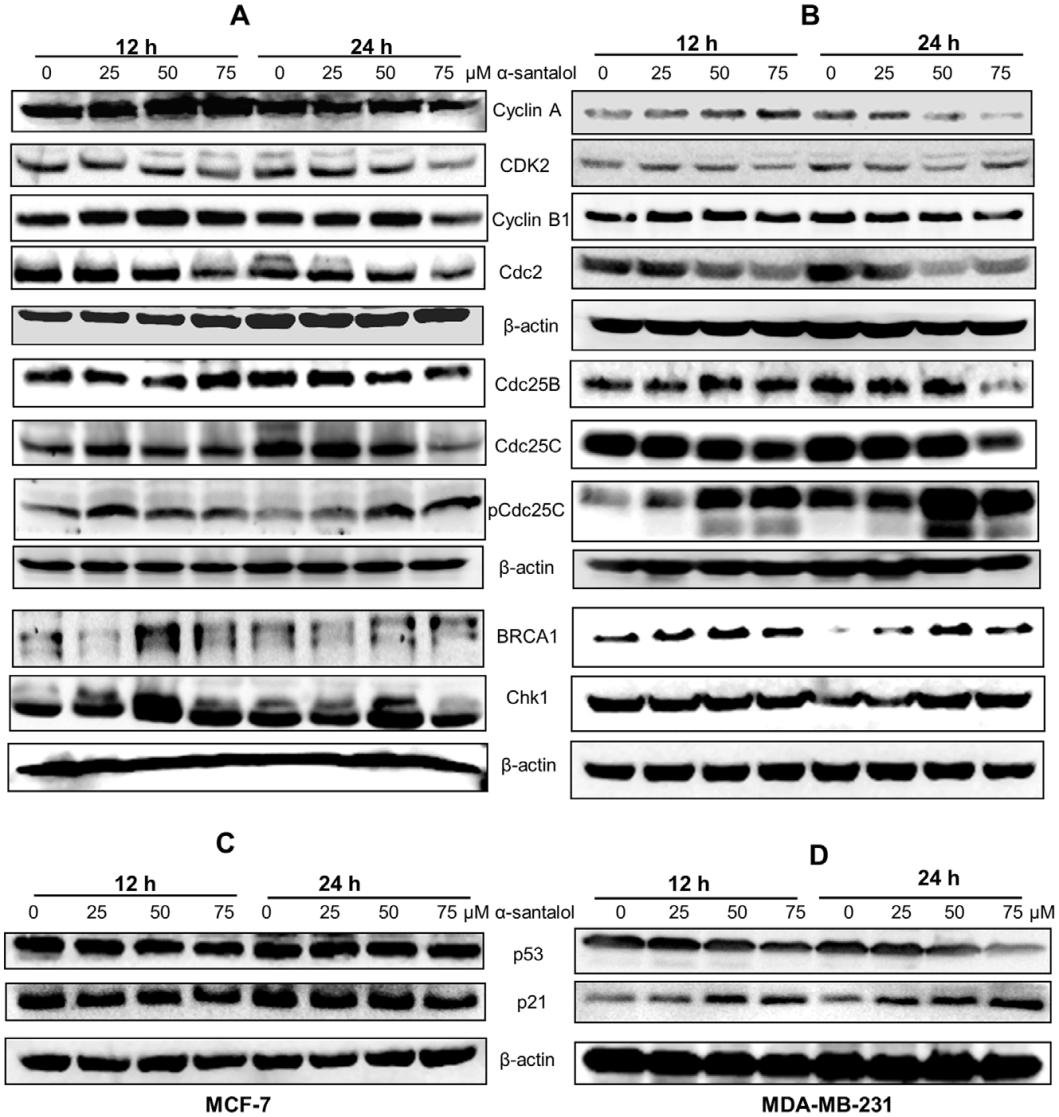
Western Blot analysis of the cell cycle regulatory proteins. (A) MCF-7 and (B) MDA-MB-231 cells were treated with α-santalol (0–75 µM) for 12 and 24 h, and equal amounts of proteins were subjected to immunoblot for the detection of the indicated G2/M regulatory proteins. (C) and (D) show the effect of α-santalol on p21 and p53 expression in MCF-7 and MDA-MB-231 cells respectively. Blots were probed for β-actin to ensure equal protein loading.

Cell cycle progression is regulated by different cyclin-dependent kinases (CDKs), which are positively regulated by cyclins and negatively regulated by CDK inhibitors at different phases [17], [18]. Since α-santalol treatment perturbed the G2/M phase of cell cycle, as assessed by DNA flow cytometry, we determined the changes in the expression of cell cycle regulatory proteins at the G2/M boundary. In MCF-7 and MDA-MB-231 cells α-santalol treatment changed the expressions of proteins involved in the G2/M phase transition as shown in Figures 7A and 7B. Cyclin A, a protein involved in S and G2/M phase was observed to be down-regulated only after 24 h treatment in both cells. The expression of CDK2, which associates with cyclin A for cell cycle progression, was also found to be decreased. Cdc2 (CDK1), that complex with cyclin A and cyclin B is essential for entry into mitosis [17], [18]. The level of Cdc2 decreased at both time points in MCF-7 and MDA-MB-231 cells. However, significant reduction in cyclin B1 was observed only at higher concentration of α-santalol after 24 h treatment. Phosphorylation of Thr 14 and Tyr 15 residues maintained CDKs in an inactive state [19]. Dephosphorylation of these residues and activation of CDKs for the G2/M transition is catalyzed by Cdc25 phosphatases such as Cdc25B and Cdc25C [19], [20]. Treatment with α-santalol resulted in a decrease in the level of Cdc25B only after 24 h treatment in MCF-7 and MDA-MB-231 cells. But a time and concentration dependent decrease in the level of Cdc25C was noticed in both cell lines. Also, the function of Cdc25C is negatively regulated by phosphorylation at Ser-216, which is essential in blocking mitotic entry [21]. α-Santalol treatment resulted in a strong increase in the level of Ser-216-phosphorylated Cdc25C in both cells.

We also tested the effect of α-santalol on BRCA1 and Chk1 expressions, which are other important factors that are involved in the G2/M regulation. In MDA-MB-231 we found an increase in BRCA1 levels at all concentrations of α-santalol, whereas in MCF-7 cells only 50 and 75 µM concentrations of α-santalol resulted in an up-regulation of BRCA1 (Figures 7A and 7B). In addition, α-santalol treatment did not produce a time or concentration dependent effect on Chk1 expression in MCF-7 cells. But, 50 µM concentrations produced a strong increase in its expression at both time points. In MDA-MB-231 cells α-santalol treatment resulted in an increase in Chk1 after 24 h treatment. Taken together, these results suggested that α-santalol might cause a reduction in the protein levels of cyclin A, CDK2, cyclin B1, Cdc2, Cdc25B, Cdc25C and an increase in pCdc25C (Ser-216) and BRCA1 in MCF-7 and MDA-MB-231 cells. These cell cycle regulators may be responsible for α-santalol induced G2/M phase arrest.

### Effect of α-Santalol on p21 and p53 Expression in MCF-7 and MDA-MB-231 Cells

We examined the levels of Cip1/p21 and p53 in α-santalol treated MCF-7 and MDA-MB-231 cells. Cip1/p21 is a CDK inhibitor and its expression is normally regulated by the tumor suppressor protein p53 [22]. As shown in Figure 7D, in MDA-MB-231 cells, the expression of mutated p53 was down-regulated and the level of CDK inhibitor p21 was up-regulated after treatment with different concentrations of α-santalol. On the contrary, wild-type p53 level and p21 level did not increase with α-santalol treatment in MCF-7 cells (Figure 7C).

## Discussion

Our laboratory has been exploring the feasibility of using α-santalol, a naturally occurring terpenoid isolated from sandalwood oil as an agent for cancer prevention and treatment. Natural products have played an important role in the development of cancer chemotherapeutic agents over the years, and almost 60% of anticancer drugs are of natural origin [23]. This study reveals that α-santalol effectively inhibits growth of human breast cancer cells which contains functional ER, and wild type p53 (MCF-7) or which lacks functional ER and contains a mutant p53 (MDA-MB-231). However, α-santalol produced relatively less toxic effect on normal breast epithelial cells, MCF-10A (Figure 1). The mode of killing that is exerted by most of the anticancer agents is by inhibition of cell proliferation through cell cycle arrest and/or induction of apoptosis, both control the outcome of chemopreventive efficacy of an agent [24]–[26].

Two major pathways of apoptosis are extrinsic or death receptor pathway and intrinsic or mitochondrial pathway. In extrinsic pathway, activation of death receptors by ligands leads to initiation of caspase cascade through the initiator caspase-8. The intrinsic pathway is characterized by the release of cytochrome c from the mitochondria and activation of the caspase cascade through initiator caspase-9 [27]. Both pathways converge to activate the executioner caspases which in turn activate cytoplasmic endonuclease, which degrades nuclear material, and proteases that degrade the nuclear and cytoskeletal proteins [27], [28]. Caspase-3, -6 and -7 functions as executioner caspases, that cleaves various substrates including PARP [28].

Our data suggests the involvement of extrinsic and intrinsic pathways of apoptosis in MCF-7 and MDA-MB-231 cells as observed by the activation of both caspase-8 and -9 in α-santalol treated cells. The executioner caspases involved in α-santalol mediated apoptosis in MDA-MB-231 cells are caspase-3 and -6, and in MCF-7cells it leads to the activation of caspases-6 and -7 (Figures 5 and 6). α-Santalol induced about five times more DNA fragmentation, which is a hallmark of late-stage apoptosis [16] in MDA-MB-231 when compared to MCF-7 (Figure 2). But in both these adherent breast cancer cell lines, we observed several cells that were detached from the plate upon α-santalol treatment. But, even after 48 h treatment, MCF-10A cells were found attached to the plate. So it could be more effective on rapidly dividing cancer cells compared to normal cells. DNA fragmentation during apoptosis is mostly mediated by the caspase-3 cleavage of DNA fragmentation factor 45 (DFF45) and release of DFF40 endonuclease which cleaves DNA at internucleosomal sites [29], [30]. Studies also suggested that only caspase-3 and -7 of all the identified caspases are essential to cleave DFF45 and result in active DFF40 [31]. So the absence of caspase-3 in MCF-7 cells used in this study and activation of csapase-7 upon α-santalol treatment could be associated with the cleavage of DEF45 and a lower percentage of DNA fragmentation in this cell line. Conflicting reports are there on the expression of caspase-3 in MCF-7 cells. Some studies reported the expression of caspase-3 and apoptosis mediated by caspase-3 in MCF-7 cells [32]–[34]. It has also been reported that MCF-7 cell line lost caspase-3 expression as a result of a 47 base-pair deletion within exon 3 of the CASP-3 gene [35]. By western blot analysis we could not find the expression caspase-3 in MCF-7 cells used in this study which suggests the absence of caspase-3. However, we detected strong activation of caspase-3 and absence of caspase-7 activation in MDA-MB-231 cells which showed a higher percentage of DNA fragmented cells. Caspase-6, which is commonly activated in both cells upon α-santalol treatment, does not play a role in DNA fragmentation [31]. Our data suggests a predominant role of caspase-3 in α-santalol mediated DNA fragmentation in breast cancer cells. Apoptosis can also occur in the absence of DNA fragmentation [36], [37]. Even in the absence of caspase-3 and lower induction of DNA fragmentation, we found strong cleavage of DNA repair enzyme PARP with disappearance of the full-length 116 kDa protein and concomitant expression of 89 kDa cleavage product in α-santalol treated MCF-7 cells. Strong cleavage of PARP was observed in MDA-MB-231 cells also (Figures 5 and 6). Cleavage of PARP during apoptosis facilitates cellular disassembly and ensures the completion and irreversibility of the process [38]. All these results clearly indicate the induction of apoptosis in ER-positive, p53 wild-type, MCF-7 and ER-negative, p53 mutated MDA-MB-231 cells by α-santalol treatment.

The development and progression of cancer is also associated with disorders in the regulation of the cell cycle in addition to loss of apoptosis [39]. The cell cycle checkpoints are frequently deranged in human tumors, causing them to enter cellular division, when conditions are not ideal and allow them to proliferate at an uncontrolled rate [18], [39]. Subsequent experiments addressed the issue whether α-santalol pre-treatment perturbs the cell cycle progression in human breast cancer cells. Flow cytometric analysis of cell cycle distribution after PI staining of DNA clearly indicated cell cycle block at G2/M phase in MCF-7 and MDA-MB-231 cells regardless of their p53 and ER status (Figures 3 and 4). However, unlike MTT and BrdU cell proliferation assays, α-santalol treatment did not show a prominent time and concentration dependent effect on the distribution of cells after 12 and 24 h treatments in both cell lines. The percentage accumulation of G2/M phase population in 75 µM α-santalol treatment was less when compared to 50 µM in both cells at both time points. This decrease in G2/M population with 75 µM concentration, and in some cases after 24 h treatment could be associated with the induction of more apoptosis at higher concentrations and longer treatment time of α-santalol.

Cell cycle progression is controlled by the activation of different cyclins and CDK complexes. Cyclin A is able to bind CDK2 and Cdc2 and promote the cell cycle progression through S and G2 phases, entry into mitosis is regulated by the activation of cyclin B/Cdc2 complex [17], [18]. In MCF-7 and MDA-MB-231 cells, α-santalol induced G2/M arrest was associated with a decrease in protein level expression of cyclin A, cyclin B1, CDK2 and Cdc2. Significant decrease of cyclin A and cyclin B1 was noticed only after 24 h treatment with higher concentrations of α-santalol. Generally, G2/M cyclins accumulate steadily during G2 and are abruptly destroyed as cells exit from mitosis and cyclin B degradation occurs at the end of mitosis [40], [41]. This could be the reason for the noticed increase of cyclin B1 expression after 12 h treatment of α-santalol. CDKs are inactivated by phosphorylation at Thr 14 and Tyr 15, dephosphorylation of these residues and activation of CDKs for cell cycle progression is controlled by members of the Cdc25 phosphatase family [19], [20]. Cdc25B and Cdc25C play an important role in G2/M transition. Cdc25B dephosphorylate and activate CDK2/cyclin A and Cdc2/cyclin B, whereas, Cdc25C dephosphorylates and activates Cdc2/cyclin B mitotic kinase complex and thereby permits cell entry into mitosis [20], [42]. Under normal conditions and after DNA damage or incomplete replication, the Cdc25C is phosphorylated on a serine residue at position 216, a mechanism through which the cells block mitotic entry [21]. Phosphorylation at Ser-216 creates a binding site for 14-3-3 family of proteins, which prevents its nuclear accumulation that is required for activation of the Cdc2/cyclin B complex in the nucleus [21]. The treatment of MCF-7 and MDA-MB-231cells with α-santalol resulted in a decrease in the levels of Cdc25B and Cdc25C, accompanied by strong increase of pCdc25C (Ser216) (Figure 7). In addition, we found an over expression of BRCA1 in α-santalol treated MCF-7 and MDA-MB-231 cells. The BRCA1 breast cancer tumor-suppressor plays an important role in DNA repair and genome stability and is also involved in G2/M checkpoint [43], [44]. BRCA1 is essential for activating the Chk1 that regulates DNA damage-induced G2/M arrest. It negatively regulates the Cdc25 family of phosphatases [44]. The down-regulation or up-regulation of the above mentioned G2/M regulatory proteins on α-santalol treatment could have led to G2/M cell cycle arrest in MCF-7 and MDA-MB-231 cells.

The tumor suppressor p53 acts as a cell cycle checkpoint regulator, contributing to cell cycle arrest in the G1 and G2 phases by a multiple pathways [22], [39]. In p53-dependent G2/M phase arrest, activation of p53 interacts with response elements present on the promoter region of p21 to increase expression of p21, which subsequently interacts with CDKs to affect cell cycle arrest [39]. The p21 can also be activated through p53-indepedent pathway [45]. Since MDA-MB-231 cells are p53 mutated, the up-regulation of p21 that we observed by α-santalol treatment in these cells may be mediated through a p53-independent pathway. Interestingly, the wild-type p53 and p21 level did not increase with treatment of α-santalol in MCF-7 cells, also suggesting p53-independent pathway. Previous studies from our laboratory on skin cancer cells by knockdown of either p21 or wild-type p53 did not change G2/M phase arrest caused by α-santalol treatment in p53 mutated A431 and p53 wild-type UACC-62 cells [15]. This suggested that α-santalol induced G2/M phase arrest independently of p21 and p53.

In conclusion, our studies reveal potential mechanisms for the antineoplastic effects of α-santalol on breast cancer cells regardless of their estrogen receptor or p53 status. The anticancer effects of α-santalol on breast cancer cells are mediated through cell cycle arrest at G2/M phase and induction of apoptosis through extrinsic and intrinsic pathways. Our laboratory is investigating the transdermal and transmammary application of α-santalol for the prevention and treatment of breast cancer in animal models. α-Santalol is relatively nontoxic to normal tissues and it has a pleasant fragrance, this will improve patient compliance and minimize undesirable systemic side effects unlike other chemopreventive/chemotherapeutic agents. α-Santalol could be effective for the prevention and treatment of ER-positive and ER-negative breast cancer. Further investigation of its activity in animal models will help to better elucidate the role for this agent in the chemoprevention of breast cancer.
