# Correction: Antineoplastic Effects of α-Santalol on Estrogen Receptor-Positive and Estrogen Receptor-Negative Breast Cancer Cells through Cell Cycle Arrest at G2/M Phase and Induction of Apoptosis

**DOI:** 10.1371/annotation/c732480c-eb97-4eff-acb6-300797f4efa9

**Published:** 2013-06-18

**Authors:** Sreevidya Santha, Ajay Bommareddy, Brittny Rule, Ruth Guillermo, Radhey S. Kaushik, Alan Young, Chandradhar Dwivedi

In the author contributions section, Sreevidya Santha (SS) should be listed as one of the authors having "Conceived and designed the experiments." 

